# PTPD: predicting therapeutic peptides by deep learning and word2vec

**DOI:** 10.1186/s12859-019-3006-z

**Published:** 2019-09-06

**Authors:** Chuanyan Wu, Rui Gao, Yusen Zhang, Yang De Marinis

**Affiliations:** 1 School of Control Science and Engineering, Shandong University, Jingshi Road, Jinan, 250061 China; 2 Diabetes and Endocrinology, Lund University, Malmo, 20502 Sweden; 3 School of Mathematics and Statistics, Shandong University at Weihai, Weihai, 264209 China

**Keywords:** Therapeutic peptide, Deep learning, Word2vec

## Abstract

***:**

Background In the search for therapeutic peptides for disease treatments, many efforts have been made to identify various functional peptides from large numbers of peptide sequence databases. In this paper, we propose an effective computational model that uses deep learning and word2vec to predict therapeutic peptides (PTPD).

***:**

Results Representation vectors of all *k*-mers were obtained through word2vec based on *k*-mer co-existence information. The original peptide sequences were then divided into *k*-mers using the windowing method. The peptide sequences were mapped to the input layer by the embedding vector obtained by word2vec. Three types of filters in the convolutional layers, as well as dropout and max-pooling operations, were applied to construct feature maps. These feature maps were concatenated into a fully connected dense layer, and rectified linear units (ReLU) and dropout operations were included to avoid over-fitting of PTPD. The classification probabilities were generated by a sigmoid function. PTPD was then validated using two datasets: an independent anticancer peptide dataset and a virulent protein dataset, on which it achieved accuracies of 96% and 94%, respectively.

***:**

Conclusions PTPD identified novel therapeutic peptides efficiently, and it is suitable for application as a useful tool in therapeutic peptide design.

## Background

Cancer continues to a burden worldwide and its frequency is expected to double in the coming decades [1]. Available treatment regimens include radiation therapy, targeted therapy, and chemotherapy, all of which are often accompanied by harmful side effects and result in high financial costs for both individuals and society [2, 3]. Anticancer peptides (ACPs) provide a new cost-efficient approach to cancer treatment, have minimal side effects, and have been shown to be promising in the treatment of various tumours by targeting mitochondria or membranolytic mechanisms [4]. Although progress has been made in preclinical applications of peptide-based methods against cancer cells, the mechanism behind the success of ACP treatments are still elusive. It is therefore highly important to be able to efficiently identify ACPs in both cancer research and drug development purposes. Due to the high costs and lengthy process of identifying ACP experimentally, various computational models have been developed to identify ACPs from peptide sequences. These advances include iACP development by g-gap dipeptide component (DPC) optimization [5, 6], and SAP peptide identification by 400-dimensional features with g-gap dipeptide pruned by the maximum relevance-maximum distance method [7]. In addition, various types of amino acid compositions (AACs) of peptide sequences have been introduced to develop prediction models such as Chou’s pseudo amino acid composition (PseAAC) [8], combinations of AACs, average chemical shifts (acACS) and reduced AAC (RAAC) [6], pseudo g-Gap DPC, amphiphilic PseAAC, and reduced amino acid alphabet (RAAAC) [9]. Other methods include computational tools developed based on the q-Wiener graph indices for ACP predication [10]. In addition, machine learning methods were adopted to promote model efficiency [6, 9, 11]. Several models have utilized support vector machine (SVM) and random forest (RF) machine learning methods [11, 12], combinations of the quantitative outcomes of individual classifiers (RF, K-nearest neighbor, SVM, generalized neural network and probabilistic neural network) [9], or a pool of SVM-based models trained by sequence-based features [13].

Novel computational models based on machine learning have also been applied to identify virulent proteins in infection pathophysiology. Virulent proteins consist of a diverse set of proteins and are important for host invasion and pathogenesis. Drug resistance to bacterial pathogens has created an urgent need to identify novel virulent proteins that may facilitate drug target and vaccine developments. Several computational models have been developed to identify virulent proteins. The first methods were developed based on similarity search methods such as the Basic Local Alignment Search Tool (BLAST) [14] and Position-specific Iterated BLAST (PSI-BLAST) [15]. Machine learning algorithms for predicting virulent proteins have also been reported that apply SVM-based models based on AAC and DPC [16], an ensemble of SVM-based models trained with features extracted directly from amino acid sequences [17], a bi-layer cascade SVM model [18], and a model based on an SVM and a variant of input decimated ensembles and their random subspace [19]. Studies have also focused on conducting feature extraction of sequences such as protein presentations, by using amino acid sequence features and evolutionary information of a given protein [19]. Moreover, a computational tool based on the q-Wiener graph indices was also proposed to effectively predict virulent proteins [10]. Despite substantial progress, identifying specific peptides from massive protein databases remains challenging.

**Fig. 1:**
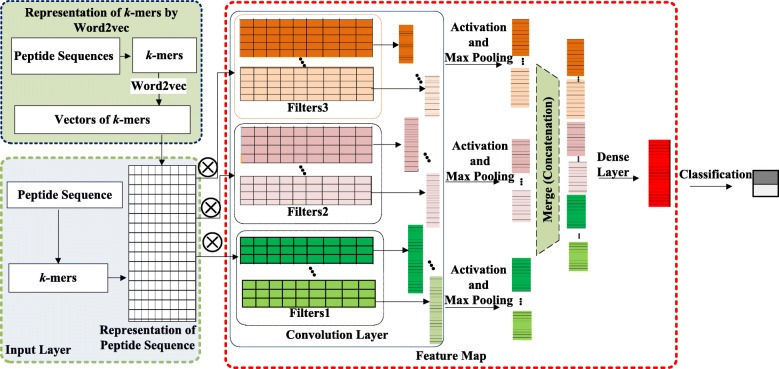
Flowchart of PTPD

To date, deep learning applications have been successful in numerous fields other than medicine, including image classification and recognition [20–22], object detection [23, 24], scene recognition [25], character recognition [26], sentence classification [27], chromatin accessibility prediction [28] and so on. Inspired by these successful deep learning applications, we propose a novel computational model called PTPD, which is based on deep learning, to identify ACPs and virulent proteins from peptide sequences (Fig. 1). To verify the efficiency of our approach, we also performed ACP and virulent protein prediction on publicly available datasets [12, 18, 29]. Our results show that PTPD is able to identify ACPs and virulent proteins with high efficiency.

## Methods

### Datasets

The ACP datasets were extracted from publicly available resources [12, 29]. A total of 225 validated ACPs from the AMPs dataset and the database of Anuran defence peptides (DADP) [30] were used as positive samples, while 2,250 randomly selected proteins from the SwissProt protein database were used as negative samples. This dataset was used to build the model. An alternative dataset and two balanced datasets were employed to evaluate the model. To compare our methods with other existing methods, we also obtained an independent dataset (i.e. Hajisharifi-Chen (HC)) from a previous study [12]. The HC dataset, which contains 138 ACPs and 206 non-ACPs, was also employed to develop prediction models in [31, 32].

The virulent protein datasets were obtained from VirulentPred [18] and NTX-pred method [16]. We adopted the SPAAN adhesins dataset, which contains 469 adhesion and 703 non-adhesion proteins, to build the PTPD model for virulent protein prediction. The neurotoxin dataset was applied as an independent dataset to evaluate the model. It contains 50 neurotoxins (positive samples) and 50 non-virulent proteins (negative samples) obtained by the NTX-pred method [16].

### Representation of *k*-mers by word2vec

Each peptide sequence was divided into *k*-mers by windowing method as previously described in [33, 34]. To represent the *k*-mers, we used the publicly available word2vec tool, which creates high-quality word embedding vectors according to a large number of *k*-mers.

**Fig. 2:**
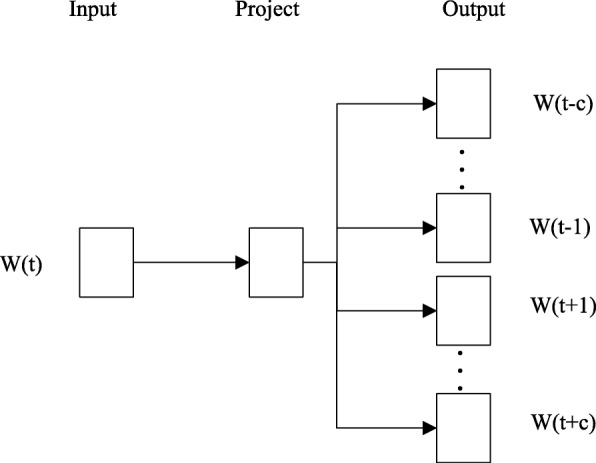
Skip-gram model structure

The word2vec tool computes vector representations of words and has been widely applied in many natural language processing tasks as well as other research applications [35–38]. Two learning algorithms are available in word2vec: continuous bag-of-words and continuous skip-gram. These algorithms learn word representations to help to predict other words in the sentence. The skip-gram model in word2vec trains the word vectors of each word based on the given corpus. Given a word (*W*(*t*)) in a sentence, skip-gram can predict the probabilities *P*(*W*(*t*+*i*)|*W*(*t*)) of nearby words *W*_*i*_(*t*−*k*≤*i*≤*t*+*k*) based on the probability of the current word *W*(*t*). Each word vector reflects the positions of the nearby words, as illustrated in Fig. 2. The goal of the skip-gram model is to maximize the following value:

1 $$  E=\frac{1}{n}\sum\limits_{t=1}^{n}{\left(\sum\limits_{-k\le i\le k,i\ne 0}{{{log}_{2}}P(W(t+i)|W(t))} \right)},  $$

where *k* denotes the size of the window, and *W*(*t*+*i*)(−*k*≤*i*≤*k*) denotes *k* words near the current word *W*(*t*), and *n* denotes the number of words.

Because word2vec can reflect the positional relationships of words in a sequence and preserve structural information, we treated the *k*-mers as the words. Using word2vec, the word embedding vector of each *k*-mer with 100 dimensions was obtained.

### Input layer

After constructing the word representation of all the *k*-mers, we mapped the peptide sequence to numeric vectors. First, we used stride *st* to divide a peptide sequence *S* with length *L*_0_ into *k*-mers of length *k*. The number of *k*-mers and the subsequent number of vectors varied because the peptide sequences (*S*) had different original lengths (*L*_0_). The vectors for one peptide were set to be the same length *L*-the length of the longest vector for those peptide sequences. Vectors with lengths shorter than *L* zero-padded at the end as in the natural language process. Finally, the peptide sequence was converted to a vector $\tilde {S}$ by the word vectors with dimensions *L*×100.

2 $$  {{\tilde{S}}_{L\times 100}}=padding({{f}_{map}}(k\_mer({{S}_{{{L}_{0}}}}))).  $$

To prevent over-fitting and to improve model generalization, dropout was applied to a fraction of the inputs (i.e., a portion of the inputs was randomly set to zero).

### Feature map

To extract features, a set of one-dimensional convolution filters was adopted to process the vectors of peptide sequences. The convolution kernel was a shape kernel with a size of (*c*×100). We used three types of convolution filters with sizes of three, four, and five. All the kernels performed convolutions on the entire representation vector. For example, using one convolution kernel with a size of (*c*×100), the feature map was constructed as follows:

3 $$  {{F}_{c}}={{[f(m)]}_{(L-c+1)\times 1}},  $$

4 $$  \begin{aligned} &f(m)=g(W\otimes \tilde{S}_{m}+b)\\ &=ReLU(\sum \limits_{i=0}^{c}{ \sum \limits_{j=0}^{100}{w(i,j)\times \tilde{s}(m+i,j)+b)}},\\ \end{aligned}  $$

where *f*(*m*) denotes the *m*th element of the feature map, *ReLU* denotes the rectified linear unit (ReLU) activation function, *w*(*i, j*) denotes the weight of the convolution kernel compiled by training, *c* denotes the size of filter, and $\tilde {S}_{m}$ denotes the *m*th block of the representation vector of the peptide sequence. ReLU [39] was used to set the negative results of the convolution calculation to zero, and is defined as follows:

5 $$  ReLU(a)=max(0,a)=\left\{ \begin{aligned} 0,&~\text{if }a \le \text{0},\\ a, &~\text{otherwise}. \end{aligned} \right.  $$

Multiple filters were used for each filter type. Let *nc* be the number of convolution filters, we applied

6 $$  {{\tilde{F}}_{c}}={{[F_{c}^{1},F_{c}^{2}\ldots,F_{c}^{nc}]}_{(L-c+1)\times nc}}.  $$

To reduce the spatial dimensions of the feature maps, max pooling was adopted following a convolution operation. A max pooling layer with a pooling window of size 2×1 and a stride of 2 was defined by the function

7 $$  \begin{aligned} &{{Z}_{c}}=({{z}_{i,j}})=pool({{{\tilde{F}}}_{c}})\\ &=[\max {{{\tilde{F}}}_{c}}(:,1),\dots,\max {{{\tilde{F}}}_{c}}(:,j),\dots,\max {{{\tilde{F}}}_{c}}(:,nc)], \end{aligned}  $$

where

8 $$  \max {{\tilde{F}}_{c}}(i,j)=\underset{i'\in [i,i+2]}{\mathop{\max }}\,{{\tilde{F}}_{c}}({{i}^{'}},j).  $$

The results were finally merged concatenated as follows:

9 $$  F{{A}_{m}}=[Z_{c1},Z_{c2},Z_{c3}],  $$

where *c*1=3, *c*2=4, and *c*3=5 denote the three filter sizes we used. Then *FA*_*m*_ was processed by a fully connected hidden layer to produce *FM*=*ReLU*(*FA*_*m*_*W*_*ft*_), where *ReLU* represents a rectified linear activation unit, and *W*_*ft*_ is the weight matrix of the fully-connected layer.

### Classification

The last layer of PTPD adopted a fully-connected layer to obtain a single output. A sigmoid activation function was set to generate the output probability between zero and one, which was defined as

10 $$  \operatorname{Sigmoid}(x)=\frac{1}{1+{{e}^{-x}}}.  $$

### Loss function and optimizer

A binary cross entropy loss function was used to train the model. The model was trained with the RMSprop optimizer. The binary cross entropy loss function between the predictions and targets was defined as

11 $$  L({{y}_{i}},{{\hat{y}}_{i}})={{y}_{i}}log({{\hat{y}}_{i}})+(1-{{y}_{i}})log(1-{{\hat{y}}_{i}}).  $$

The total cost of the two classes was

12 $$  L=\sum\limits_{i=1}^{2}{L({{y}_{i}},}{{\hat{y}}_{i}}).  $$

### Model evaluation

The performance of PTPD was evaluated by various metrics, including the sensitivity (Sn), specificity (Sp), prediction accuracy (Acc), Matthew’s correlation coefficient (MCC), and the area under the curve (AUC) of the receiver-operating characteristic (ROC) curve. These metrics were defined as follows:

13 $$  \left\{ \begin{array}{l} Sn=\frac{TP}{TP+FN} \\ Sp=\frac{TN}{TN+FP} \\ Acc=\frac{TP+TN}{TP+TN+FP+FN} \\ MCC=\frac{(TP\times TN)-(FP\times FN)}{\sqrt{(TP+FP)(TP+FN)(TN+FP)(TN+FN)}} \\ \end{array} \right.,  $$

where *TP* denotes true positives, *TN* denotes true negatives, *FP* denotes false positives, *FN* denotes false negatives.

## Results

### Model performance

To verify the proposed method, we executed the proposed model on ACPs and virulent protein datasets. Each dataset was randomly divided into three groups. The first group, which consisted of 75% of the complete dataset, was used to train the model. The second group of data, 15% of the entire dataset, was used to minimize over-fitting. The third group, 10% of the entire dataset, was used to evaluate the performance of the trained PTPD model. For ACP identification, the performance of PTPD was first measured using the test data from the main dataset, and then further tested on an alternative dataset. Furthermore, we also evaluated the performance of PTPD on two types of balanced datasets (Table 1).

**Table 1:** Performance of PTPD on the ACP dataset

Dataset	Sn(%)	Sp(%)	Acc(%)	MCC	AUC
ACP main dataset	99.90	86.60	98.50	0.92	0.99
ACP alternative dataset	96.20	86.70	94.80	0.80	0.97
ACP balanced dataset 1	100	86.20	93.10	0.87	0.99
ACP balanced dataset 2	94.20	86.20	90.20	0.81	0.97
HC dataset	100	83.00	94.00	0.87	0.99

PTPD achieved high performance scores of Sn = 94.2%, Sp = 86.2%, Acc = 90.2%, Mcc = 0.8, and AUC = 0.97, respectively. Moreover, to evaluate the generalizability or robustness of the prediction model, we executed PTPD on the independent HC dataset, as shown in Table 1. The AUCs of the five data sets were all higher than 0.97. Thus, PTPD offers stable performance even on unbalanced data sets (Table 1).

To evaluate the performance of PTPD, we conducted an evaluation on the test data of the SPAAN adhesins dataset. We also tested the performance of PTPD on an independent Neurotoxins dataset (Table 2).

**Table 2:** Performance of PTPD on the virulent protein dataset

Dataset	Sn(%)	Sp(%)	Acc(%)	MCC	AUC
SPAAN adhesins dataset	95.60	73.3	88.2	0.70	0.94
Neurotoxins dataset	98.00	94.00	96.00	0.92	0.93

The five performance metrics (Sn, Sp, Acc, MCC, and AUC) achieved by PTPD on the virulent protein dataset are higher than 95.6%, 73.3%, 88.2%, 0.7, and 0.93, respectively, which confirms the good performance of PTPD. Sp on the SPAAN adhesins dataset had a relatively lower value (Table 2).

### Comparison with the state-of-the-art methods

For verification purposes, we compared the proposed method with other state-of-the-art methods on the identification of ACPs and virulent proteins on two independent datasets.

#### Comparison performed on independent aCP dataset

**Fig. 3:**
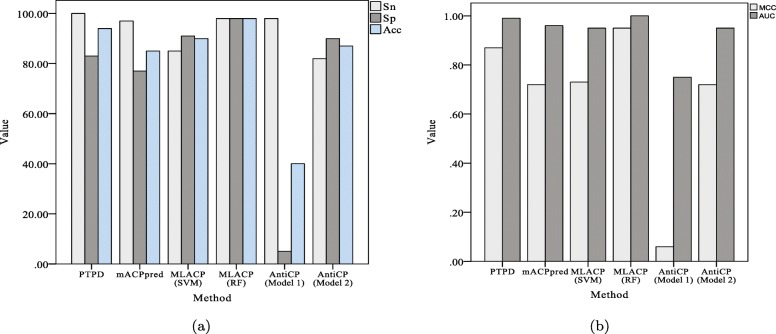
Comparison of different methods on the HC dataset. **a** Sn, Sp and Acc of different methods. **b** MCC and AUC of different methods. Sn: the sensitivity; Sp: the specificity; Acc: the prediction accuracy; MCC: Matthew’s correlation coefficient; AUC: the area under the curve of the receiver-operating characteristic curve

To further evaluate the performance of PTPD to predict ACPs, we compared its performance with those of some state-of-the-art methods (i.e., AntiCP [29], MLACP [12], and mACPpred [40]) on an independent HC dataset (Table 3 and Fig. 3). PTPD performed equally as well as MLACP (RF) on the HC dataset. The proposed PTPD has the highest sensitivity, relatively higher AUC, ACC, and MCC, and intermediate specificity. Thus, PTPD offers relatively better generalizability on independent datasets than do the other tested state-of-the-art methods for identifying ACPs.

**Table 3:** Comparison of PTPD with state-of-the-art methods on the HC dataset

Method	Sn(%)	Sp(%)	Acc(%)	MCC	AUC
PTPD	100	83.00	94.00	0.87	0.99
mACPpred [40]	97.00	77.00	85.00	0.72	0.96
MLACP (SVM)[12]	85.00	91.00	90.00	0.73	0.95
MLACP (RF)[12]	98.00	98.00	98.00	0.95	1.00
AntiCP (Model 1)[29]	98.00	5.00	40.00	0.06	0.75
AntiCP (Model 2)[29]	82.00	90.00	87.00	0.72	0.95

#### Comparison performed on an independent virulent protein dataset

**Fig. 4:**
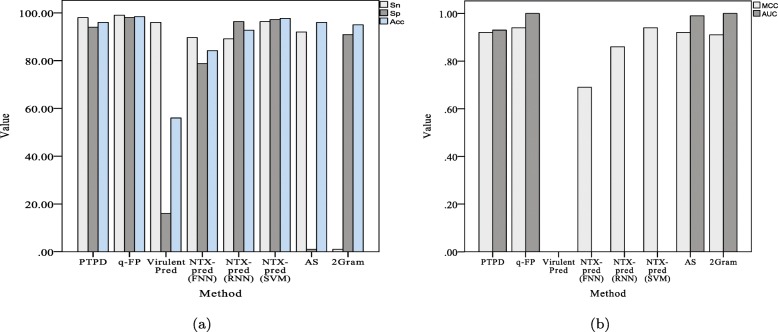
Comparison of different methods on the neurotoxin virulent proteins dataset. **a** Sn, Sp and Acc of different methods. **b** MCC and AUC of different methods. Sn: the sensitivity; Sp: the specificity; Acc: the prediction accuracy; MCC: Matthew’s correlation coefficient; AUC: the area under the curve of the receiver-operating characteristic curve

We also compared the performance of PTPD with that of q-FP [10], AS and 2Gram [41], VirulentPred [18], and NTX-pred [16] on a bacterial neurotoxins dataset (Table 4 and Fig. 4).

**Table 4:** Comparison of PTPD with state-of-the-art methods on the Neurotoxins dataset

Method	Sn(%)	Sp(%)	Acc(%)	MCC	AUC
PTPD	98.00	94.00	96.00	0.92	0.93
q-FP [10]	99.03	98.00	98.40	0.94	1
VirulentPred [18]	96.00	16.00	56.00	-	-
NTX-pred(FNN) [16]	89.65	78.78	84.19	0.69	-
NTX-pred(RNN) [16]	89.12	96.35	92.75	0.86	-
NTX-pred(SVM) [16]	96.32	97.22	97.72	0.94	-
AS [41]	92.00	1.00	96.00	0.92	0.99
2Gram [41]	1.00	90.91	95.00	0.91	1

Again, the overall performance of PTPD was relatively better than those of other methods. Thus, we can conclude that PTPD is able to predict potential virulent proteins with high accuracy.

### Parameter settings

**Fig. 5:**
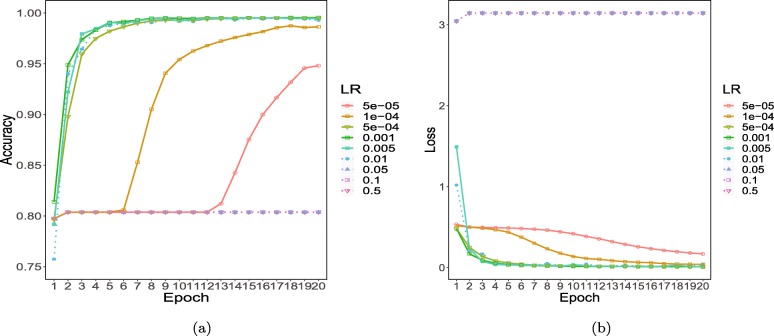
Performances under different learning rates: **a** accuracy under different learning rates; **b** loss under different learning rates

Because model convergence is related to the learning rate, we set the learning rate variously to 0.5, 0.1, 0.05, 0.01, 0.005, 0.001, 0.0005, 0.0001, 0.00005, and 0.00001 for ACP training. The accuracy and loss values under the different learning rates are shown in Fig. 5.

The model achieved its highest accuracy (98.5%) and the lowest loss (0.03) when the learning rate was set to 0.0001, which was subsequently selected for model training. The detailed parameter settings are shown in Table 5.

**Table 5:** Parameter setting

Parameters	Value
Number of kernels	150,150,150
Filter size	3,4,5
*k*-mer dimensions	100
Batch size	100
Epoch	20
Learning rate	0.0001

## Discussion

The model performance presented in this study suggests that PTPD possesses good generalizability and robustness. The comparison between PTPD and other methods showed that PTPD outperformed the other tested state-of-the-art methods for independent data analysis.

The performance of PTPD benefits from several major factors: (1) word2vec was applied to extract representation vectors of *k*-mers to consider the co-existence information of *k*-mers in peptide sequences. (2) For the feature map, a convolution neural network (CNN) architecture was used to automatically extract features without domain experts. (3) Dropout and max-pooling operations were adopted to avoid over-fitting.

## Conclusions

Identifying new ACPs and virulent proteins is an extremely labour-intensive and time-consuming process. In this paper, we proposed a computational model based on deep learning that predicts therapeutic peptides with in a highly efficient manner. We then present a new deep learning-based prediction model that achieves better recognition performances compared to those of other state-of-the-art methods. We first trained a model to extract feature vectors of all *k*-mers using word2vec. Next, the peptide sequences were converted into *k*-mers, and each peptide sequence was represented by the vectors compiled by word2vec. The CNN then automatically extracted features without expert assistance, which decreases the reliance on domain experts for feature construction. The CNN was configured with three types of filters, and dropout and max-pooling operations were applied to avoid over-fitting. After fusing the features, ReLU activation was used to replace any negative values in the output of the CNN layer with zeros. Finally, the sigmoid function was used to classify the peptide.

The performance and generalizability of PTPD were verified on two independent datasets. The trained model achieved AUCs of 0.99 and 0.93, respectively, which confirmed that the proposed model can effectively identify ACPs and virulent proteins.

In summary, the PTPD model presented in this paper outperformed other tested methods. Nevertheless, the approach still suffers because the inability to assign values regarding which features are most important for identifying favourable bioactivity. In future studies on potential structures and feature selection methods, we may consider other available network architectures such as generative adversarial networks. Some new methods that have been successfully applied to natural language processes might also facilitate further research. Our study confirmed that PTPD is an effective means for identifying and designing novel therapeutic peptides. Our approach might be extensible to other peptide sequence-based predictions, including antihypertensive [42, 43], cell-penetrating [44], and proinflammatory [45].

## Data Availability

The datasets supporting the conclusions of this article are available for ACP datasets from [12, 29] and for Virulent protein from [16, 18].

## Abbreviations

AAC: Amino acid composition
Acc: Accuracy
ACP: Anticancer peptide
AUC: The area under ROC curve
CNN: Convolution neural networks
DADP: Defence peptide
FN: False negative
FP: False positive
MCC: Matthew’s correlation coefficient
Pse-g-Gap DPC: Pseudo g-Gap dipeptide composition
PseAAC: Pseudo amino acid composition
PSI-BLAST: Position specific iterated BLAST
PSSM: Position Specific Scoring Matrices
PTPD: Prediction of therapeutic peptide by deep learning and word2Vec
RAAAC: Reduced amino acid alphabet
RAAC: Reduced amino acid composition
ReLU: Rectified linear unit
RF: Random Forest
SAP: Sequence-based model
Sn: Sensitivity
Sp: Specificity
SVM: Support vector machine
TN: True negative
TP: True positive
